# Increased physical activity frequency in primary school adolescents is related to reduced risk of self-reported adverse mental health symptoms

**DOI:** 10.3389/fpubh.2024.1506432

**Published:** 2024-12-11

**Authors:** Chunmei Li, Xiong-Zhe Han

**Affiliations:** ^1^Normal College of Yanbian University, Jilin, China; ^2^College of Physical Education, Yanbian University, Jilin, China

**Keywords:** adolescents, physical activity, psychological health, adverse mental health symptoms, risk factor

## Abstract

**Purpose:**

The sedentary lifestyle and mental health issues of primary school students are major public health issues in China and globally. Some studies have shown that regular physical activity is beneficial to health, but there are few epidemiological investigations on the relationship between physical activity and psychological problems. The purpose of this study is to explore the relationship between physical activity and mental health.

**Materials and methods:**

We used data from our study, which was based on the Yanbian University School of Physical Education health survey of primary education, in which 822 elementary school students in grades 4–6 participated. Physical activity is evaluated through three questions (intensity, frequency, and duration), and mental health issues are evaluated through SPSS.

**Results:**

In our study, 488 primary school students (200 males and 288 females) participated and completed self-reported data on physical activity and mental health variables. The findings revealed that a higher frequency of physical activity was significantly associated with decreased risks of various mental health symptoms. Specifically, students who reported a higher frequency of physical activity exhibited a lower risk of depressive symptoms (RR = 0.31, 95% CI = 0.14–0.71, *p* < 0.05), anxiety (RR = 0.35, 95% CI = 0.18–0.67, *p* < 0.05), low self-esteem (RR = 0.48, 95% CI = 0.26–0.90, *p* < 0.05), and life dissatisfaction (RR = 0.58, 95% CI = 0.35–0.96, *p* < 0.05). The risk of somatic complaints reporting was slightly lesser if the prevalence of physical activity reported at screening was higher (RR = 0.93, 95% CI = 0.50–1.76), although this result was not statistically significant (*p* > 0.05). These findings highlight the importance of regular physical activity in promoting mental health among primary school students.

**Conclusion:**

Given the dose–response relationship between poor mental health and lack of physical activity, it is necessary to actively promote primary school students to participate more actively in physical activities.

## Introduction

With the rapid advancement of socioeconomic conditions and the evolution of medical paradigms, the significance of mental health in shaping lifelong happiness and achievements has become increasingly evident, elevating mental health concerns to the forefront of societal discussions. However, the mental wellbeing of Chinese children and adolescents remains a cause for concern. In tandem with China’s swift socioeconomic progress, adolescents have faced an array of challenges, including employment, education, familial issues, romantic relationships, interpersonal conflicts, financial pressures, pornography, and violence, all contributing to varying degrees of distress. Consequently, psychological issues among teenagers have proliferated, with incidents stemming from psychological and behavioral problems occurring intermittently. Phenomena such as smoking, alcoholism, drug abuse, societal misconduct, teenage pregnancy, suicide, violent crimes, and more continue to surface. Notably, these issues extend beyond teenagers, impacting primary and secondary school students alike.

National and international research has underscored the pivotal role of physical activity in enhancing mental health. Physical activity serves as a bulwark against the emergence of negative emotions and mitigates the risk of mental illness (1). Studies have demonstrated that engagement in sports regulates physiological changes within the body, effectively reducing stress levels induced by the external environment and buffering stress responses. This, in turn, alleviates the adverse effects of stress on mental health, elevates mood, and fosters positive outcomes (2–4).

Physiologically, exercise augments the levels of neurotransmitters like 5-hydroxytryptamine, dopamine, and norepinephrine in brain tissue, exerting antidepressant and anxiolytic effects, thereby inducing feelings of happiness. Furthermore, physical activity not only boosts these neurotransmitter levels but also enhances the functionality of brain neurotransmitters, including the release of serotonin and endorphins. This suggests that exercise elicits a euphoric sensation, operating through a mechanism akin to SSRI antidepressants (5, 6). Practically, exercise diverts patients’ attention and aids in alleviating depression or anxiety. Regularly active students confront stress and anxiety with greater positivity and exhibit robust resilience against frustration (7). Additionally, physical activity guards against chronic diseases such as cardiovascular disease and type 2 diabetes (8) and has a profound impact on mental health issues, particularly depression (9, 10). For adolescents, physical exercise profoundly influences their self-development, openness, and affinity, demonstrating significant improvement effects. Moreover, it enhances their psychological tolerance and self-control. Physical activity is invaluable in combating physiological and psychological fatigue, fostering a cooperative spirit, and nurturing a competitive mindset.

In recent years, the global population affected by mental disorders, such as anxiety and depression, has surged from nearly 10% to now accounting for 30% of the global burden of non-fatal diseases (11). The incidence of mental health issues escalates dramatically during adolescence (12, 13), with anxiety and depression being the primary drivers of disease burden and impairment among young people (14). These conditions often manifest as fatigue, lack of energy, and appetite loss (15). A study on long-term trends in anxiety and depression symptoms among Icelandic adolescents between 1997 and 2006 reported an increase in symptoms and a higher frequency of consultations with psychologists, psychiatrists, and social workers (8). Anxiety and depression are potent predictors of adverse health and psychosocial outcomes, including behavioral problems, academic difficulties, substance abuse, suicide attempts, and low self-esteem (14). Furthermore, adolescents with anxiety and depression are at a significantly heightened risk of developing these disorders in adulthood (14). Hence, identifying risk and preventive factors during adolescence is a crucial public health priority.

Adolescence is a unique life stage characterized by rapid physical and mental growth, acquisition of knowledge and physical strength, making mental health particularly crucial. The World Health Organization’s health standards stipulate that individuals must possess not only physical health but also psychological wellbeing, coupled with good social adaptation, to be deemed healthy. Therefore, the mental health of adolescents is essential for their better social adaptation, enabling them to learn and work more effectively, and harness their wisdom and abilities in these domains. Despite compelling evidence linking mental health and physical activity, few large-scale epidemiological studies have delved into the nature of this relationship. Given these considerations, the present study aims to meticulously investigate the correlation between the intensity, frequency, and duration of physical activity and mental health issues among primary school students.

## Methods

### Research population

The Student Mental Health Study was a large-scale health survey involving 822 students in grades 4–6 from elementary schools in a city. Of these, 630 students agreed to participate, aged between 10 and 12 years old, with 53% boys and 47% girls. A total of 192 students did not participate due to lack of interest in the study or absence on the survey day. The assessment was conducted from mid-December through early February 2019. Participants completed the survey electronically through an online platform that provided background, health, and lifestyle information pertinent to the survey topic. The questionnaire covered age, gender, participation in physical activity, frequency of vigorous exercise per week, symptoms of mental health problems (such as anxiety, depression, and somatic complaints), self-esteem, and satisfaction with life. Written informed consent was obtained from both the participants and their guardians.

The psychological questionnaire was completed in a quiet and private environment at school to ensure that students could express their feelings truthfully without external interference. The sessions were scheduled during students’ break time or after-school hours, supervised by professionally trained research assistants or class teachers, to ensure standardization and confidentiality of the questionnaire completion. We also emphasized to the students the anonymity of the questionnaire and the confidentiality of the results to alleviate any psychological burden they might feel. All studies were conducted with strict confidentiality and with the approval of an Ethics Committee.

### Definition of physical activity

Refers to sports such as walking, running, swimming, jumping rope and ball games. Physical activity was evaluated using three indicators: average frequency of exercise per week, average intensity of exercise, and average duration of exercise. Average intensity and average duration (16). (1) “What is the average frequency of your physical activity per week? (1 = never, 2 = once a week, 3 = twice a week, 4 = three times a week, 5 = 4–5 times a week, 6 = almost every day); and (2) “If you do this type of exercise regularly, once or more than once a week: what is the intensity of your exercise? (1 = I take it easy and do not sweat or get out of breath; 2 = I let myself sweat a little and breathe as well as I can; 3 = I let myself get out of breath and sweat; 4 = I let myself get to the point of near depletion); and (3) “How long does each workout last? (1 = <15 min, 2 = 15–29 min, 3 = 30–45 min, 4 = 45 min to 1 h, 5= >1 h”). For analysis purposes, the variable duration per exercise was recorded into these two categories: less time = “less than 30 min duration per exercise” and more time = “less than 30 min duration per exercise.” According to the International Physical Activity Guidelines, adolescents and children should be involved in healthy physical activity at least 3 days/week (17). For the purpose of analysis, the variable average number of physical activities per week was recorded into these two categories: less frequent = “<4 times per week” and high frequent = “4 or more times per week.”

### Physical activity

Objective measurement of physical activity using an accelerometer (ActiGraph, model GT3X+, Manufacturing Technology Inc., Pensacola, FL, USA). An accelerometer measures the change in acceleration over time. The output variable is the count of each time interval, processed through the filtering and summary process, and expressed as the count of each time interval “epoch.” The Settings used in this study were a sampling frequency of 30 Hz and a 10-s epoch. Get the average count and number of steps per hour. It has been reported that assessment with an accelerometer can effectively estimate physical activity.

### Research indicators

Using the Hopkins Symptom Checklist (SCL-90), mental distress was evaluated, which includes a 10-item anxiety subscale and a 15-item depression subscale to assess mental health. Each item is estimated on a five-point Likert scale ranging from 1 (“Almost never”) to 5 (“almost always”). Thresholds based on the possible scores’ median were used to define healthy and unhealthy scores, with 30 for depressive symptoms, 12 for anxiety symptoms, and 24 for somatic discomfort and complaints. This SCL-90 rating scale was previously employed in an Icelandic study on physical activity and mental health among university students, using the same cut-off points (18).

### Covariates

Physical indicators were selected as covariates in conjunction with prior research. Previous studies have identified relationships between physical indicators (19–21), physical activity (21, 22) and mental health (23–25). The collected data have demonstrated that the two are correlated.

### Body metrics

The weight (kg) and height (m) of the testers were weighed by the organization of the student’s school unit and the overall body and composition of regional soft tissue was counted by dual-energy X-ray absorptiometry (DXA). By Lunar bone densitometer (GE Healthcare, Madison, WI, USA) to obtain the percentage of body fat and by an established registered radiologist located at the institution, all DXA scans were performed using the same equipment in the same hospital.

### Accelerometer wearing

The triaxial accelerometer used was the ActiGraph® Monitor (Model GT3X; ActiGraph, Pensacola, CA, USA) (46 × 33 × 15 mm; weight 19 g, additional technical features). The accelerometer measures acceleration and deceleration in three spatial dimensions according to a vertical vector (*x*), an anteroposterior vector (*y*), and a mediolateral vector (*z*). The vector magnitude (VM) was calculated as follows: VM = (*x*^2^ + *y*^2^ + *z*^2^). The epoch interval for the accelerometer was set at 1 s. A computer was used to initialize and synchronize the accelerometer. Participants who recorded less than 10 h of activity per day were excluded from the analyses. PA levels were categorized as follows: sedentary activity, 0–180 counts. 15 s^−1^; light activity, 181–757 counts. 15 s^−1^; moderate activity, 758–1,112 counts. 15 s^−1^; and vigorous activity, >1,112 counts. 15 s^−1^. The interinstrument reliability of this device is reported to be better for moderate and vigorous activities than for sedentary activity. Data were averaged and expressed in counts.min^−1^.

### Statistical analysis

Continuous variables were explicited as mean and standard deviation, and categorical variables were explicited as frequency and percentage. The t-test was employed for gender variations in continuous variables and the chi-square test was employed for gender variations in categorical variables. Pearson’s correlation analysis was employed to assess the relationship among the main variables of interest. Poisson regression analyses were used to compute relative risks (RR) and 95% confidence intervals (CIs) for reporting anxiety, depression, low self-esteem, somatic complaints and life dissatisfaction symptoms. Depressive symptoms, anxiety, somatic complaints, low self-esteem, and life dissatisfaction associated with self-reported frequency of vigorous exercise and objectively measured total exercise and exercise duration. First, separate Poisson regressions were conducted among each of the independent variables, subjective vigorous exercise and objective overall exercise and each mental health consequences. All Poisson regression models were modified for the following potential confounders: gender, percent body fat. *p* < 0.05 was regarded as a remarkable variations or relationship. Statistical analyses were carried out by SPSS statistical software version 25.0.

## Results

In this study, we used accelerometers to obtain free-living physical activity data for participants. Participants were asked to wear accelerometers all day on five regular school days and two weekend days. Details of how long the accelerometer is worn, how often the data is collected, and how the data is processed and analyzed are described in the Methods section. To ensure data quality, we have taken the following measures: Participants are given detailed guidance and training before the data collection begins to ensure that they wear the device correctly and understand the importance of data collection. We also implement rigorous data integrity checks to ensure that the data collected is complete and reliable. During the analysis, we excluded any data segments with no activity recorded for 15 min or more in a row to reduce the impact of invalid data. Ultimately, 445 participants met the criteria for valid measurements of free-living physical activity (data were complete for five regular school days and two weekend days), and 440 of these participants also completed self-reported data on physical activity and mental health variables. The final study sample therefore included 440 student participants, 180 of whom were male and 260 of whom were female.

The average body mass index (BMI) for girls was 20.3 kg/m^2^, with an overweight prevalence (BMI ranges from 25 to 30 kg/m^2^) of about 13% and an obesity prevalence (BMI of 28.5 kg/m^2^) of about 3%. These figures are remarkably greater for girls compared to boys (19.5 kg/m^2^). The average percentage of body fat for girls was 28.5%, again remarkably greater than for boys (16.7%) and statistically significant at *p* < 0.0001 (Table 1).

**Table 1:** Basic information on the subject of the study.

	Total (*n* = 488)	Males (*n* = 200)	Females (*n* = 288)	*p*-value
Age, years, mean (SD)	11.8 (0.3)	11.8 (0.3)	11.9 (0.3)	0.07
BMI, kg/m^2^, mean (SD)	19.9 (3.1)	19.5 (2.8)	20.3 (3.2)	0.06
Normal (BMI: 20 < *x* ≤ 25), *n* (%)	386 (79.4)	166 (83.8)	220 (76.4)	
Overweight (BMI: 25 < *x* ≤ 30), *n* (%)	26 (10.7)	7 (7.1)	19 (13.2)	
Obese (BMI: 30 *x*), *n* (%)	10 (2.1)	2 (1.0)	8 (2.8)	
Underweight (BMI: *x* ≤ 20), *n* (9%)	38 (7.8)	16 (8.1)	22 (7.6)	
Body fat, %mean (SD)	22.3 (9.0)	16.7 (6.6)	28.5 (6.3)	<0.0001
Vigorous PA, subjective, *n* (%)				0.12
Less (<4x/week)	174 (35.7)	60 (30.0)	114 (39.6)	
More (>4x/week)	314 (64.3)	140 (70.0)	174 (60.4)	
Total PA’, objective, cpm/day, mean (SD)	2,039 (472)	1,993 (452)	2,071 (484)	0.2
Below median (*N* = 122; 53 males, 69 females)	1,673 (241)	1,650 (244)	1,691 (239)	0.34
Above median (*N* = 122; 47 males, 75 females)	2,404 (345)	2,380 (289)	2,420 (378)	0.53
Depression				
Score, mean (SD)	17.6 (8.9)	14.4 (6.3)	19.7 (9.8)	<0.0001
Symptoms (score 30), *n* (%)	26 (10.7)	4 (4.0)	22 (15.3)	<0.005
Anxiety				
Score, mean (SD)	6.6 (3.6)	5.3 (2.5)	7.6 (4.0)	<0.0001
Symptoms (score 12), *n* (%)	34 (13.9)	3 (3.0)	31 (21.5)	<0.0001
Somatic complaints				
Score, mean (SD)	15.6 (6.6)	13.1 (5.2)	17.3 (7.0)	<0.0001
Symptoms (score 24), *n* (%)	35 (14.3)	8 (8.0)	27 (18.8)	0.02
Self-esteem				
Score, mean (SD)	213 (6.8)	22.1 (7.0)	20.7 (6.7)	0.11
Low self-esteem (score 15), *n* (%)	38 (15.6)	12 (12.0)	26 (18.1)	0.2
Life satisfaction				
Score, mean (SD)	26.1 (7.1)	26.1 (7.7)	26.1 (6.6)	0.99
Life dissatisfaction (score ≤ 20), *n* (%)	48 (19.7)	24 (24.0)	24 (16.7)	0.16

In Tables 2, 3, an association among self-reported exercise intensity, duration of exercise and physical activity with self-reported mental health conditions, i.e., depression, anxiety and somatic symptoms (Table 2) and self-esteem and life dissatisfaction (Table 3). After correcting for potential confounders (gender, percent body fat), reporting higher frequency of physical activity was associated with decreased levels of depressive symptoms (19.7% vs 5.7%, RR = 0.31, 95% CI = 0.14–0.71), anxiety (25.3% vs 7.6%, RR = 0.35, 95% CI = 0.18–0.67), low self-esteem (24.1% vs 10.8%, RR = 0.48, 95% CI = 0.26–0.90) and life dissatisfaction (28.7% vs 14.7%, RR = 0.58, 95% CI = 0.35–0.96) risk associated. The associated risk of somatic complaints reporting was slightly lesser if the prevalence of physical activity reported at screening was higher (16.1% vs 13.4%, RR = 0.93, 95% CI = 0.50–1.76), but this result was not associated with self-reported, objectively measured intensity and duration of physical activity.

**Table 2:** Associations of physical activity with symptoms of depression, anxiety, and somatic complaints.

	Depressive symptoms		Anxiety symptoms		Somatic complaints	
	Yes, *N* (%”)	RRP (95%CI)	Yes, *N* (%”)	RR (95%CI)	Yes, *N* (%”)	RR (95%CI)
Vigorous PA, subjective						
Less (4x/week)	17 (19.5)	Ref.	44 (25.3)	Ref.	28 (16.1)	Ref.
More (4x/week)	18 (5.7)	0.31 (0.14, 0.71)*	24 (7.6)	0.35 (0.180.67)**	42 (13.4)	0.93 (0.50, 1.76)
Total PA, objective						
Below median	22 (9.0)	Ref.	28 (11.5)	Ref.	34 (13.9)	Ref.
Above median	30 (12.3)	1.24 (0.60, 2.56)	40 (16.4)	1.38 (0.742.54)	36 (14.8)	0.99 (0.53, 1.85)
Total duration						
Below median	14 (5.7)	1.33 (0.74, 2.76)	24 (9.8)	1.44 (1.23, 2.54)	26 (10.7)	1.55 (1.29, 2.03)
Above median	38 (15.6)	Ref.	44 (18.0)	Ref.	44 (18.0)	Ref.

PA, Physical activity.

**Table 3:** Associations of physical activity with low self-esteem and life dissatisfaction.

	Low self-esteem		Life dissatisfaction	
	Yes, *N* (%”)	RR (95%CI)	Yes, *N* (%”)	RRP (95%CD)
Vigorous PA, subjective				
Less (<4x/week)	42 (24.1)	Ref.	50 (28.7)	Ref.
More (≥4x/week)	34 (10.8)	0.48 (0.26, 0.90)	46 (14.7)	0.58 (0.35, 0.96)
Total PA, objective				
Below median	46 (18.9)	Ref.	54 (22.1)	Ref.
Above median	30 (12.3)	0.64 (0.35, 1.19)	42 (17.2)	0.84 (0.51, 1.39)
Total duration				
Below median	18 (7.4)	1.31 (1.15, 0.66)	24 (9.8)	1.38 (1.20, 1.72)
Above median	29 (23.8)	Ref.	72 (29.5)	Ref.

## Discussion

In this large 2019 citywide health survey, which included students in elementary schools, we obtained that prevalance of physical activity was negatively related to mental health problems and that more frequent physical activity was related to a lesser risk of reporting depression, anxiety, and depressive symptoms. We found that participation in vigorous exercise at least four times per week reduced the reduction of risk (42–69%) for a variety of mental health problems (excluding somatic complaints). Previous studies, regarding the extent to which exercise intensity affects the mental health of primary and secondary school students have varied, which may be due to variations in the participants age and the methods used to assess exercise and mental health (26). Our study showed little relationship between exercise intensity and duration and mental health. This is broadly in line with the explorations of Trinh L and Feng Q et al. (27, 28). However, in a survey of adolescents in Iran, comfort in life was associated with self-reported physical activity (29). It has also been confirmed that quality of life and satisfaction are related to both self-reported exercise intensity and frequency of physical activity (26). Another study showed that anxiety and depression were not correlated to the prevalance of physical activity, but at the same time negatively correlated with measures of duration and intensity of physical activity (30).

Our study showed that self-reported duration and intensity of physical activity were minimally correlated with psychological problems, a result that is certainly surprising. We hypothesize that intensity and duration of exercise may depend on the exercise frequency and that self-perception while exercising may have a greater impact on mental health. Some reviews have suggested that high- and low-intensity physical activity shows signs of benefit for adolescent mental health, yet the difference is not large (31–33). However, studies have concluded (26). Interestingly, in another article on interventions of exercise to decrease or prevent anxiety, exercise intensity had little effect on depression and anxiety scores in elementary and middle school students (34). The limitation of our study was that we could not objectively collect exercise-related data from the students, but only their subjective exercise information.

For many years, adolescent mental health has been a public issue that cannot be ignored. On the one hand, we can dynamically assess mental health problems and psychosocial outcomes in adolescents through anxiety and depression, and on the other hand, we can predict an individual’s risk of developing psychological disorders in adulthood (35). It has been noted that physical activity can have a positive moderating effect on the psyche through physical mechanisms (36). Specifically, physical activity can ameliorate mental health issues like anxiety and depression by modulating neurotransmitter levels and body composition (28, 37, 38). In addition, physical activity can ameliorate psychological problems and promote mental health through social communication and interaction as well as increased self-efficacy (39–42).

The limitations of the study primarily revolve around two key aspects. Firstly, the sample size utilized in the research is somewhat limited, which may impact the generalizability of the findings. To address this, plans are in place to collect additional samples in future studies to conduct a more robust sensitivity analysis. Secondly, while the study acknowledges the importance of sleep duration, sleep quality, age, and adolescence as critical factors in mental health research, it may not have fully accounted for the intricate ways in which these variables interact. Given that numerous studies have highlighted the significance of these factors, subsequent research should include a broader range of observed variables to enhance the sensitivity analysis and provide a more comprehensive understanding of their influence on mental health outcomes.

## Conclusion

We found that more frequent exercise leads to lower risks of depression, anxiety, and dissatisfaction with life in primary school students. Our research findings advocate that adolescents should participate in sports more frequently to achieve a healthier psychological state. We suggest that policy makers can increase the frequency and duration of youth sports to achieve better results. Meanwhile, we also look forward to more relevant clinical research focusing on this topic in the future.

## Strengths and limitations of this study

### Strengths

The sample selection is large and innovative; previous studies have mainly focused on college students, European and American groups, and so on.

### Limitations

The options set in the questionnaire are subjective and cannot reach a completely uniform standard. In addition, besides the physical variables of the testers, this study failed to exclude other potential interfering factors, such as family education background.

### Literature review

Previous studies have mainly focused on college students, European and American groups, and used questionnaire surveys to investigate their physiological status. This study focuses more on the physical and mental health of primary school students, and uses objective indicators to evaluate their physiological indicators.

## Data Availability

The original contributions presented in the study are included in the article/supplementary material, further inquiries can be directed to the corresponding authors.
